# A radiographic and histological study to compare red (650 nm) versus near infrared (810 nm) diode lasers photobiomodulation for alveolar socket preservation

**DOI:** 10.1038/s41598-024-57114-x

**Published:** 2024-03-22

**Authors:** Mohamad Anwar Abd-Elhaleem Othman, Ahmed Abbas Zaky, Elsayed Abdullah Eltayeb, Nesma Mohamed Khalil

**Affiliations:** 1 National Institute of Laser Enhanced Sciences, Cairo University, Cairo, Egypt; 2 Medical Applications of Lasers Department, National Institute of Laser Enhanced Sciences, Cairo University, Cairo, Egypt; 3 Oral Biology Department, Faculty of Dentistry, Alexandria University, Alexandria, Egypt

**Keywords:** Laser, Photobiomodulation, Extraction, Socket Healing, Alveolar Ridge Preservation, Dental implants, Dental lasers

## Abstract

Previous findings indicated that the laser photobiomodulation is more effective than the control or placebo in preserving the alveolar socket. This study aimed to compare two different lasers regarding their effectiveness in aiding alveolar socket preservation. Twenty extraction sockets were selected then divided into two equal groups. Group A was exposed to 650 nm Diode laser, and Group B to 810 nm Diode laser following the same protocol and parameters after a standard alveolar socket preservation procedure with collagen plug. Radiographic analysis with cone beam computed tomography was done to compare the alveolar bone surface area immediately after extraction and three months post-operatively, while bone samples collected before implant drilling were histologically examined for newly formed bone evaluation and histomorphometric analysis in terms of percentage of new bone surface area, percentage of unmineralized bone and finally, immunohistochemical analysis of Osteocalcin reaction surface area as well as optical density. Radiographically, infrared (810 nm) Diode effect on alveolar bone surface area has significantly exceeded the red laser, while histologically, red (650 nm) Diode has demonstrated statistical significance regarding all parameters; newly formed bone surface area percentage, unmineralized bone area percentage and finally Osteocalcin bone marker reaction surface area percentage and optical density. Under the specified conditions and laser parameters, photobiomodulation using the 810 nm Diode got the upper hand radiographically, yet histologically, the red 650 nm Diode managed to dominate all histological parameters when both employed as an adjunct to alveolar socket preservation procedures.

## Introduction

Dental extraction was, and still, one of the most relevant lines of treatments offered in dental clinics, regardless of current evolution in restorative dentistry^1–3^.

On top of reasons that necessitate dental extraction arise dental caries as well as periodontal disease, particularly in developing countries, then severe trauma comes in third place^4–6^.

Dental implants are currently extensively utilized with much success in the restoration of function, esthetics and speech in fully or partially edentulous situations^7–10^.

As a consequence of dental extraction, the alveolar ridge at the edentulous site experiences a physiological bone loss process causing a decrease in the bucco-lingual and apico-coronal dimensions. Most of the alveolar bone resorption occurs within the initial 3 to 6 months post-extraction^11–14^.

Socket preservation procedure has become the accepted standard of care for patients undergoing dental implant installation following a dental extraction^15^.

Alveolar socket preservation encompasses a wide variety of regenerative treatment modalities that have been described in the literature, including socket grafting with bone substitute, (whether it would be autograft, allograft, xenograft, or alloplast), overbuilding of the facial bony wall, socket occlusion using a barrier element, a combination of materials, or platelet rich fibrin. It is worth mentioning that primary intention healing was not of prime importance with described techniques. Several systematic reviews have well supported the effectiveness of alveolar socket preservation, hence, recommended its application in current dental practice^16^.

Socket plug technique involves occluding the extraction socket with a collagen plug on top of a bone substitute grafting material, or even alone. In former applications, it eliminates the need for complicated soft tissue grafting procedures to cover the bone graft, such as free gingival graft, or flap advancement, and allows for a flapless surgery, while in the later, it was proved to support soft and hard tissue healing in an economic, conservative and simple approach^17–19^.

Multiple systems have been proposed to classify extraction socket defects, most of them are mainly directed towards the immediate implant placement procedure^20–24^, what mainly matters in socket preservation procedures is the presence of the buccal plate, which, in case of its absence, necessitates the use of shape preserving barrier membranes in an attempt to regain the outline of bony architecture^17^.

Generally speaking, dental lasers are divided into: High-power lasers that are typically used for the purposes of ablation, cutting and coagulating of soft tissues as well as conservative cutting of hard tissues, while low-power lasers, commonly referred to as "cold lasers," are utilized in order to support tissue repair, such as muscle, nerve, joint, skin, and bone injuries by active contribution in various stages of healing mechanisms at the cellular level^25–27^.

Low-level laser therapy, also known as photobiomodulation therapy (PBMT), has been found to have a range of positive effects. These include greater lymphocytic proliferation and activation, increased phagocytosis by macrophages, increased secretion of growth factors by fibroblasts, enhanced motility of epithelial cells, increased granulation tissue, and decreased synthesis of inflammatory mediators^28,29^.

Studies conducted on animals suggest that PBMT therapy can increase the proliferation, collagen synthesis, and mineralized matrix production of osteoblastic cells; thus, leading to an increase in the amount and rate of new bone formation, as well as promoting epithelial cell proliferation and angiogenesis^30–32^.

The utilization of PBMT as an adjunct therapy for alveolar socket preservation has been universally accepted; however, the best parameters for its application remain controversial. There is no consensus in the literature regarding the correct wavelength, frequency, dose, and light intensity parameters to reduce bone loss following tooth extraction^33–35^.

The literature review has revealed that laser PBMT has been the most effective treatment for alveolar socket healing when compared to control or placebo treatments^35–44^.

Therefore, in order to ascertain the most suitable tool for photobiomodulation of extraction sockets, we have decided to conduct a study comparing the efficacy of two lasers in aiding post extraction alveolar bone repair.

## Materials and methods

### Study design

A randomized, double-blinded clinical trial.

ClinicalTrials.gov Identifier: NCT05911607 (Date: 23/06/2023).

### Study participants

**Figure 1:**
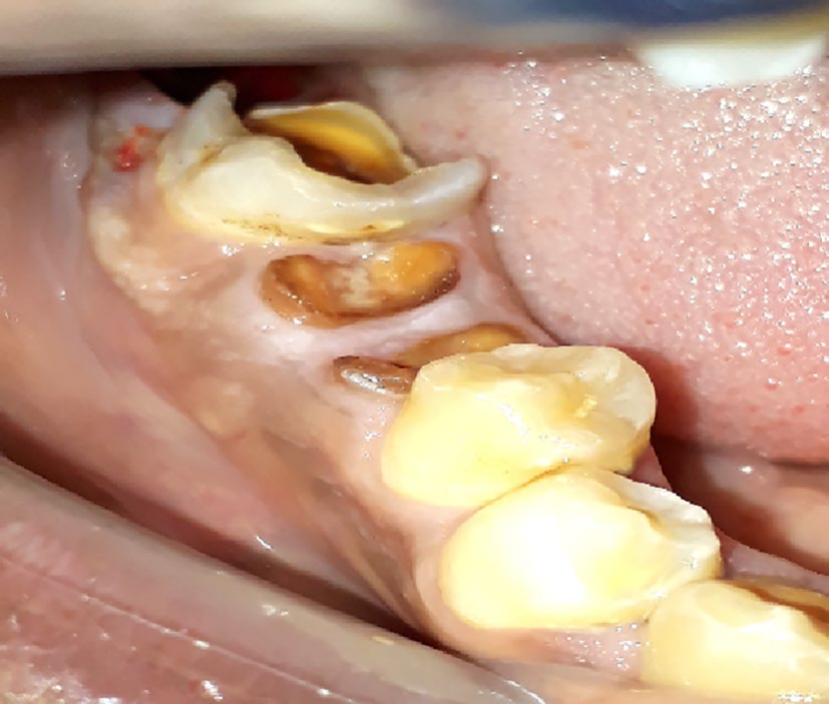
Unrestorable mandibular first molar.

Twenty patients, who had twenty mandibular posterior teeth indicated for extraction (Fig. 1) were included in the study from a Cairo University-affiliated clinic. Inclusion and exclusion criteria for selected patients were:

Inclusion criteria

- Male 35–50 years old.
- Acceptable oral hygiene (no bleeding on probing, no recession, normal gingival color and texture with stated regular brushing at least twice daily)
- Badly decayed un-restorable mandibular molar.

Exclusion criteria

- Smokers
- Metabolic disorders that may interfere with future implant osseointegration (uncontrolled diabetes, thyroid gland disorders, vitamin D deficiency).
- Any sign of active infection at the extraction site (severe pain, swelling).
- Any sign of active periodontal disease at the treatment segment (teeth mobility, gingival rescission, horizontal bone loss).
- Periodontally involved teeth.
- The use of bisphonates.
- Current or previous history of chemotherapy or radiotherapy.

All participants reviewed, agreed and signed an informed consent for inclusion ahead of participation. The study was conducted in accordance with declaration of Helsinki, in addition, the protocol was approved by the Ethics Committee of National Institute of Laser Enhanced Sciences, Cairo University: NILES – EC – CU 23/6/8.

### Sample size calculation

The minimal sample size is calculated based on a previous study aimed to evaluate the effects of Photobiomodulation (PBM) therapy on alveolar bone repair^36^. Rosero et al.^36^ reported that the PBM therapy improved the newly bone trabeculae formation and their connectivity which increased bone surface, indicating the positive effect of the laser on alveolar human socket repair. The sample size was calculated to detect the difference in surface area of newly formed bone trabeculae (primary outcome). Adopting a power of 80% (b = 0.20) to detect a standardized effect size in size in surface area of newly formed bone trabeculae (primary outcome) of 1.491, and level of significance 5% (α error accepted = 0.05), the minimum required sample size was found to be 8 patients per group (number of groups = 2) (Total sample size = 16 patients)^45,46^. After adjustment for a dropout rate of 10%, the sample size was increased to 10 patients per group (number of groups = 2) (Total sample size = 20 patients)^47^.

**Software:** The sample size was calculated using GPower version 3.1.9.2^48^.

**Sampling technique:** Convenience sampling.

### Randomization technique

A randomized, double-blinded approach was adopted to allocate participants to either Group A or Group B. The allocation sequence was generated using permuted block randomization performed by the main researcher and the block size was variable^49^. The allocation sequence was concealed using sealed opaque envelopes^50^.

**Blinding*:*** A double "full" blind strategy was used. Participants were blinded and outcome assessors were blinded to the patients' assigned groups^51^.

### Study procedure

An atraumatic extraction was performed following the administration of Articaine 4% 1:200,000 (Laboratorios Inibsa, Spain). In this regard, certain surgical techniques were followed as much as the clinical situation allows such as: avoiding buccal plate guttering, separation of roots to facilitate path of exfoliation, limiting bone removal, if any, to the inter radicular septum, and finally, using rotation movement during separated roots extraction instead of bucco-lingual luxation.

**Figure 2:**
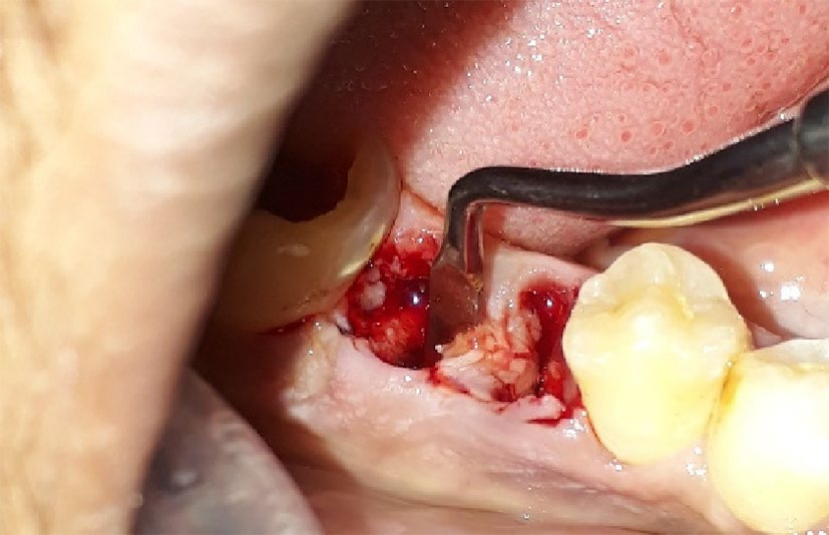
Extraction & socket curettage.

**Figure 3:**
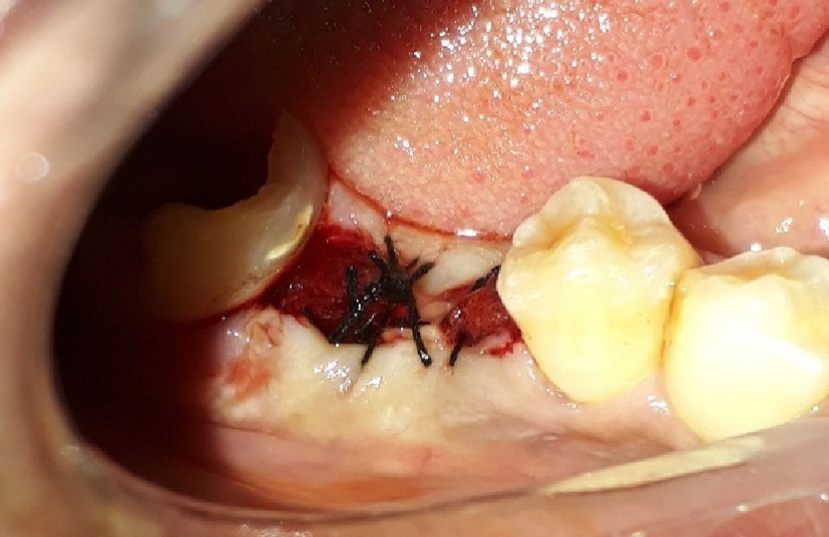
Insertion & fixation of collagen plug.

Subsequently, a standard alveolar socket preservation procedure was conducted^52^, comprising socket walls curettage (Fig. 2), followed by pure saline irrigation and the application of a collagen plug (Collacone, Botiss Biomaterials GmbH, Germany) that was secured in place with a cross suture (Silk Braided 3/0, Non Absorbable, Surgical Suture, China) (Fig. 3).

The participants were randomly split into two groups for PBM therapy (each group included 10 sockets).

**Figure 4:**
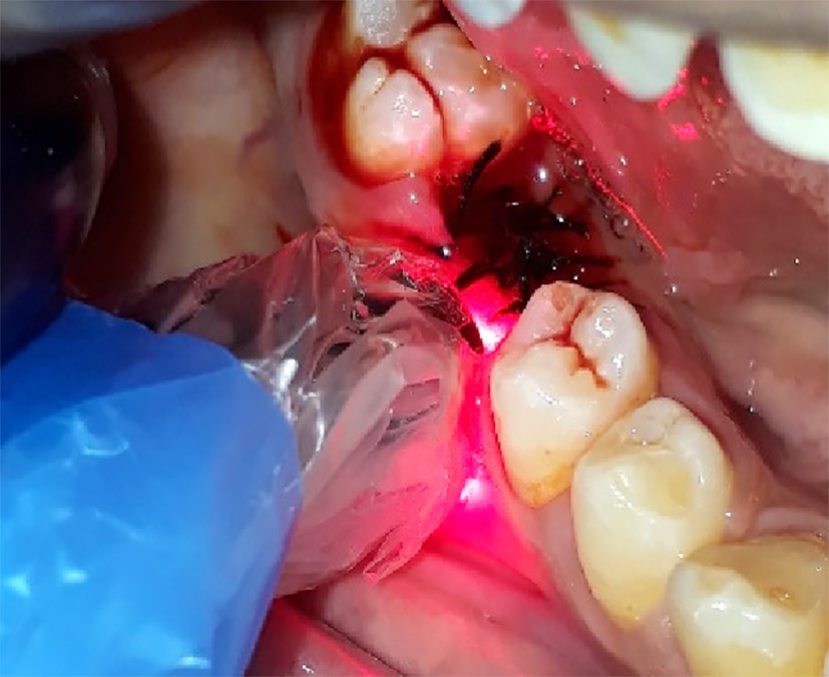
Diode 650 nm PBMT.

Group A was exposed to 650 nm Diode laser (Fig. 4).

**Figure 5:**
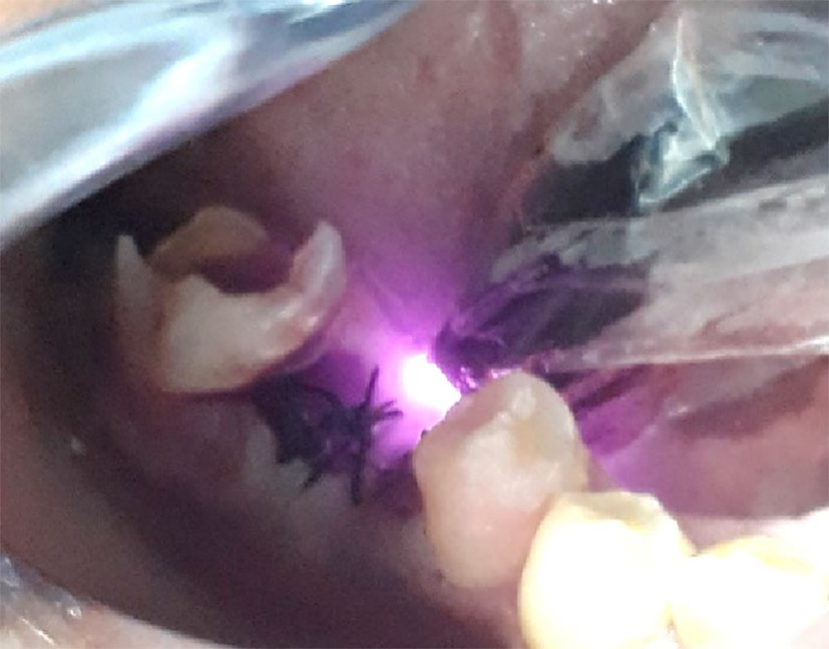
Diode 810 nm PBMT.

Group B was exposed to 810 nm Diode laser (Fig. 5).

The PBM therapy consisted of 3 surface touch mode irradiation (buccal, occlusal and lingual) each lasting for one minute. The protocol was carried out for 6 sessions at intervals of immediately post extraction, and 1, 3, 5, 7 and 15 days^36,37^. The Diode (GaAlAs) laser device used was fabricated at the Department of Laser Engineering, Higher Institute of Laser Enhanced Sciences, Cairo University, Egypt, emitting a continuous wave beam with power at 100 ml W. Table 1 shows the details of laser irradiation parameters.

**Table 1:** Photobiomodulation parameters.

Parameter	Value
Wavelength	Group (A): 650 nm Group (B): 810 nm
Power	100 mlW
Mode	Continuous
Spot Area	0.28 cm^2^
Power Density	0.35 W/cm^2^
Irradiation Time	180 s (60 s × 3 spots)
Energy Density	63 J/cm^2^

**Figure 6:**
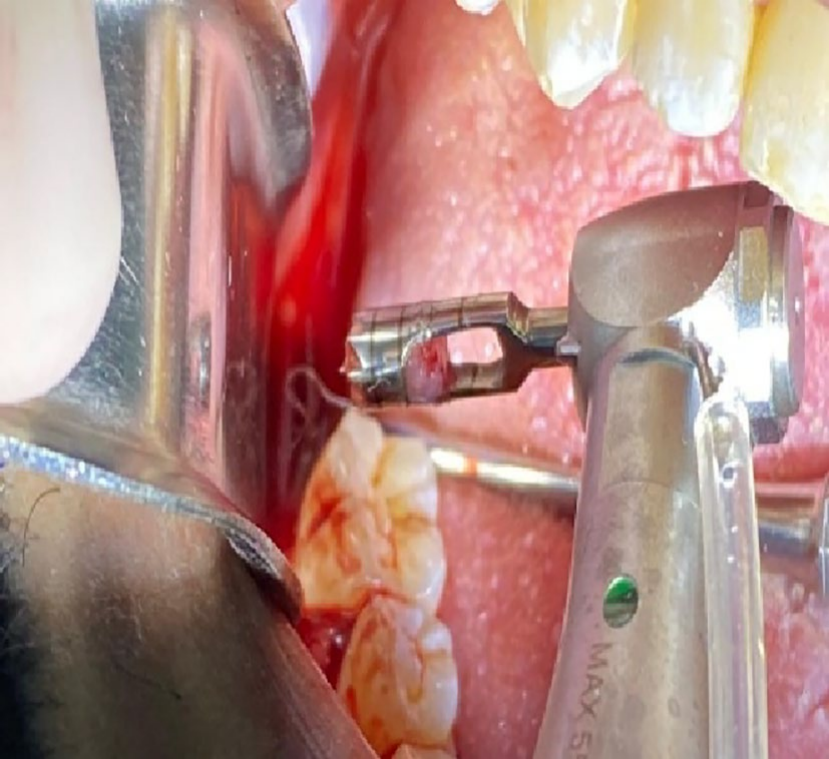
A bone sample collected from a preserved socket ahead of implant osteotomy preparation using a trephine bur.

At the 3 months mark post extraction, patients were recalled for implant placement in the preserved sockets, such procedure was planned based on the CBCT scan. After administration of local anesthesia and a head of regular implant drilling, a bone sample “for histological analysis” was collected from the middle of the edentulous ridge using a 3 × 10 mm trephine bur. (Fig. 6).

**Figure 7:**
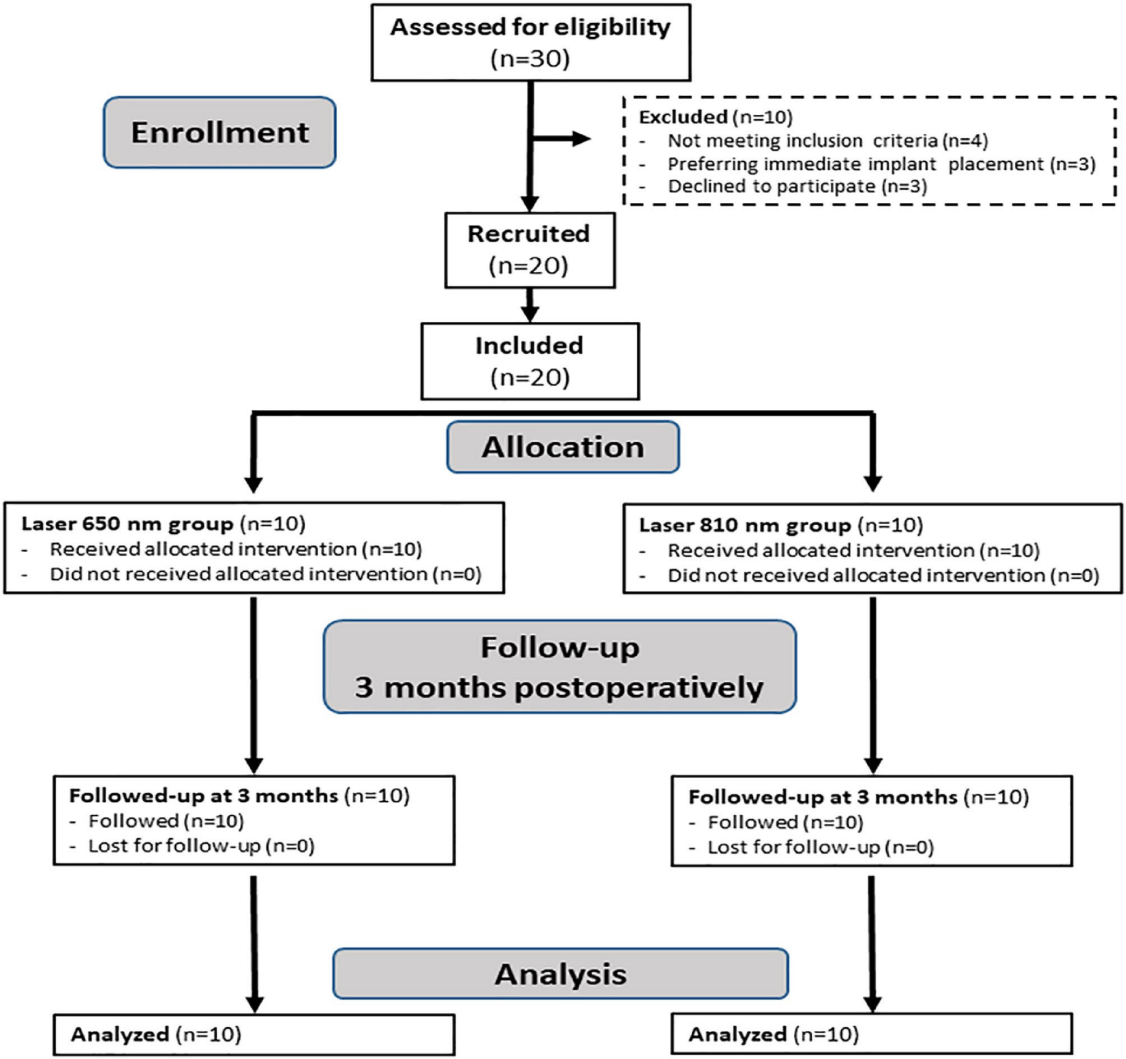
Flow of the experimental procedures based on the CONSORT guidelines.

A summarized timeline of the study procedures based on the consolidated standards of reporting trials (CONSORT) is shown in Fig. 7.

#### Radiographic evaluation

Cone beam computed tomography scans were performed for each patient twice: first just after extraction and again three months later (such scan was utilized for implant planning). Fusion module was used to compare identical coronal slices of socket sites in terms of the total surface area of the alveolar ridge on the radiograph.

For all patients at both phases, CBCT scans were performed at the same maxillofacial scanning facility using the same scanning tool (Scanora, 3Dx, Soredex, Finland).

First and second radiographs were compared using the OnDemand 3D software's fusion module (Cybermed, South Korea) by visualizing overlaid sets of DICOM data.

**Figure 8:**
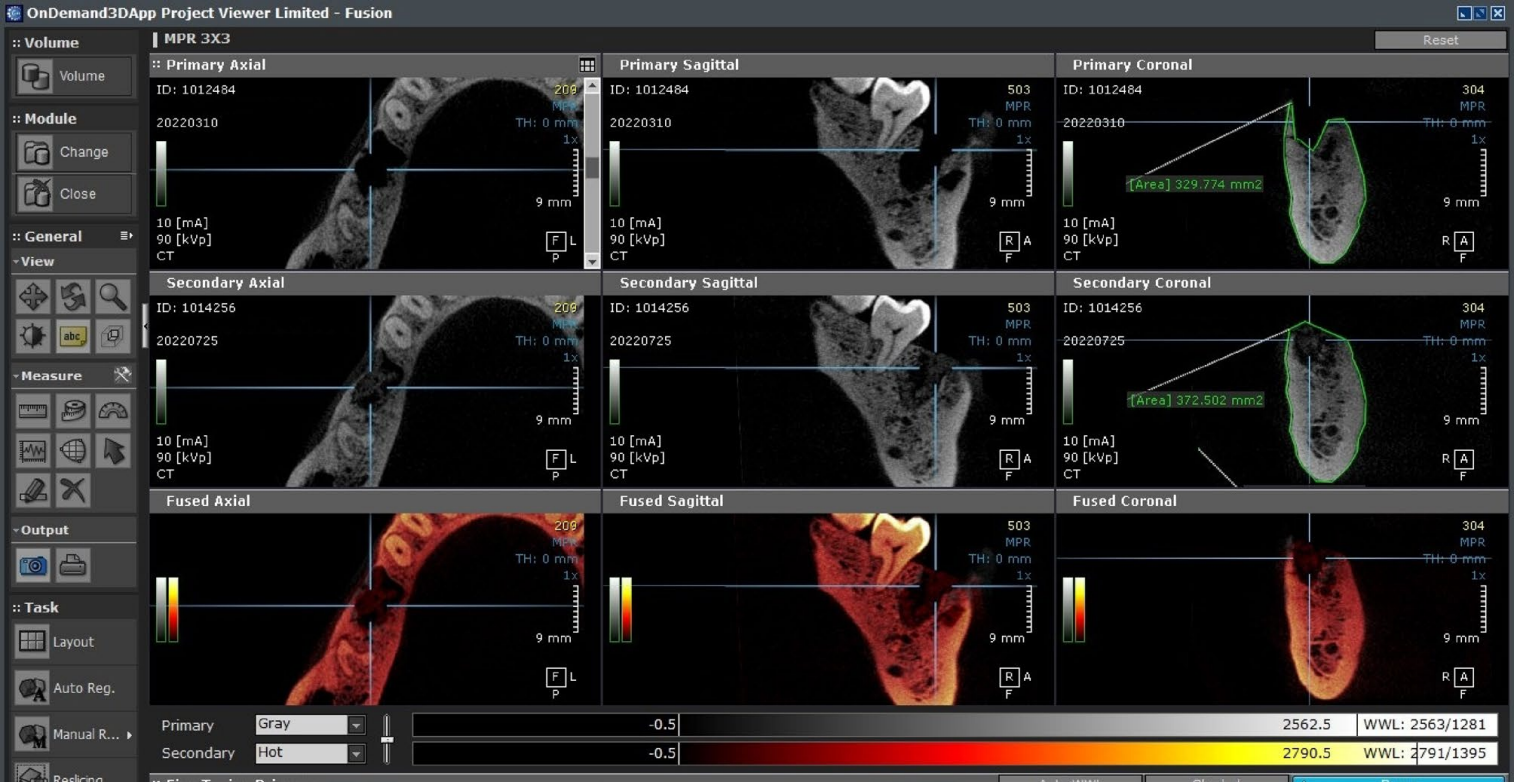
Alveolar bone border tracing measurements using OnDemand 3D software fusion mode.

Same coronal section, at the middle of each extraction site, an area measurement tool was used to trace present bone borders. (Fig. 8).

#### Histological observation

Each specimen collected at implant surgical stage was immediately fixed in 10% neutral buffered formalin, then, processed for two staining techniques; Haematoxylin & Eosin (H&E)^52^ and Masson–Goldner Trichrome^53^ stains for general examination of newly formed bone and detection of unmineralized bone respectively.

Slides were examined using Olympus BX41 Phase Contrast & Darkfield Microscope (Olympus Corporation, Japan), then, sections were captured using Olympus DP20 digital microscope camera (Olympus Corporation, Japan) for examination of histological slides, which was undertaken following a fully blinded approach within each study group.

#### Immunohistochemical testing

Immunohistochemical analysis was carried out using (Osteocalcin Antibody) at dilution of 1:200 heat mediated antigen using immunohistochemical detection system recommended by manufacturer (Wuhan Fine Biotech Co, Ltd, China)^54^.

### Histomorphometric analysis

Histomorphometric measurements were carried out using two different software’s, in 3 serial sections for each specimen and a mean value was calculated, such process was repeated for each bone sample.

- Newly formed bone surface area percentage: Using QuPath 2.0 (University of Edinburgh, Scotland) histology software, both manual and wand tracing tools were applied on the × 40 magnified field images of H&E stained specimens in the aim of accurate tracing of newly formed bone area percentage in relation to total field^55^.
- Unmineralized bone percentage: In addition, ImageJ 1.53t (National Institutes of Health, USA) software was used in color deconvolution mode on the × 40 magnified Masson–Goldner Trichrome stained specimens in order to separate field colors. Red isolated areas were calculated as representative of unmineralized bone area percentage compared to the whole tested field^56^.
- Osteocalcin bone marker expression: Finally, color deconvolution mode of ImageJ 1.53t was used once again on × 400 magnified sections. 5 non-overlapping fields were selected in each section and mean values (area percentage & mean color intensity) were calculated. This time, isolated red areas demonstrated the anti-Osteocalcin marker reaction, software was able to estimate two values; (1) reaction surface area percentage compared to the entire field besides, (2) mean color intensity which was then used to calculate optical density (indicative for marker reaction intensity) via the following formula^57^.

OD = 255/mean intensity.

Where 255 represents value of maximum intensity for 8-bit images.

## Results

### Radiographic findings: (Table 2)

**Table 2:** Surface area immediately and 3 months postoperatively in both studied groups.

Bone area (mm)	Wave length group		Test of significance *p *value
	650 nm Group (n = 10)	810 nm Group (n = 10)	
Immediate			
Min.–Max	244.48–329.77	252.17–345.96	Z_(MW)_ = 0.227
Median	292.91	287.83	*p* = 0.821 NS
95% CI of the median	269.71–305.56	271.33–334.62	
At 3 months			
Min.–Max	241.12–372.50	278.73–403.70	
Median	295.14	330.95	Z_(MW)_ = 1.512
95% CI of the median	263.87–329.62	292.79–359.89	*p* = 0.131 NS
Test of significance	Z_(WSR)_ = 1.784	Z_(WSR)_ = 2.803	
* p *Value	*p* = 0.074 NS	*p* = 0.005*	
Bone Area (percentage change) (%)			
Min.– Max	− 217 to 12.96	1.31–22.88	
Median	1.49	13.61	Z_(MW)_ = 2.570
95% CI of the median	− 0.72 to 9.15	4.03–18.99	*p* = 0.01*

n: Number of patients, Min–Max: Minimum–Maximum, CI: Confidence interval, MW: Mann–Whitney U test, WSR: Wilcoxon Signed Ranks Test, *: Statistically significant (*p* < 0.05), NS: Statistically not significant (*p* ≥ 0.05).

Both groups presented comparable measurements immediately post extraction (*p* = 0.821), which is deemed statistically insignificant, same as 3 months post-operative dimensions, no statistical significance was found across both groups (*p* = 0.131). However, what really matters was the fact that, within each study group, comparing immediately post extraction and 3-month post-operative measurements, a statistical significance was documented in favor of Group (B) (810 nm) (*p* = 0.01), denoting the relative advantage of such laser in regard to alveolar bone surface area increase.

### Histological observation

#### H & E stained sections

**Figure 9:**
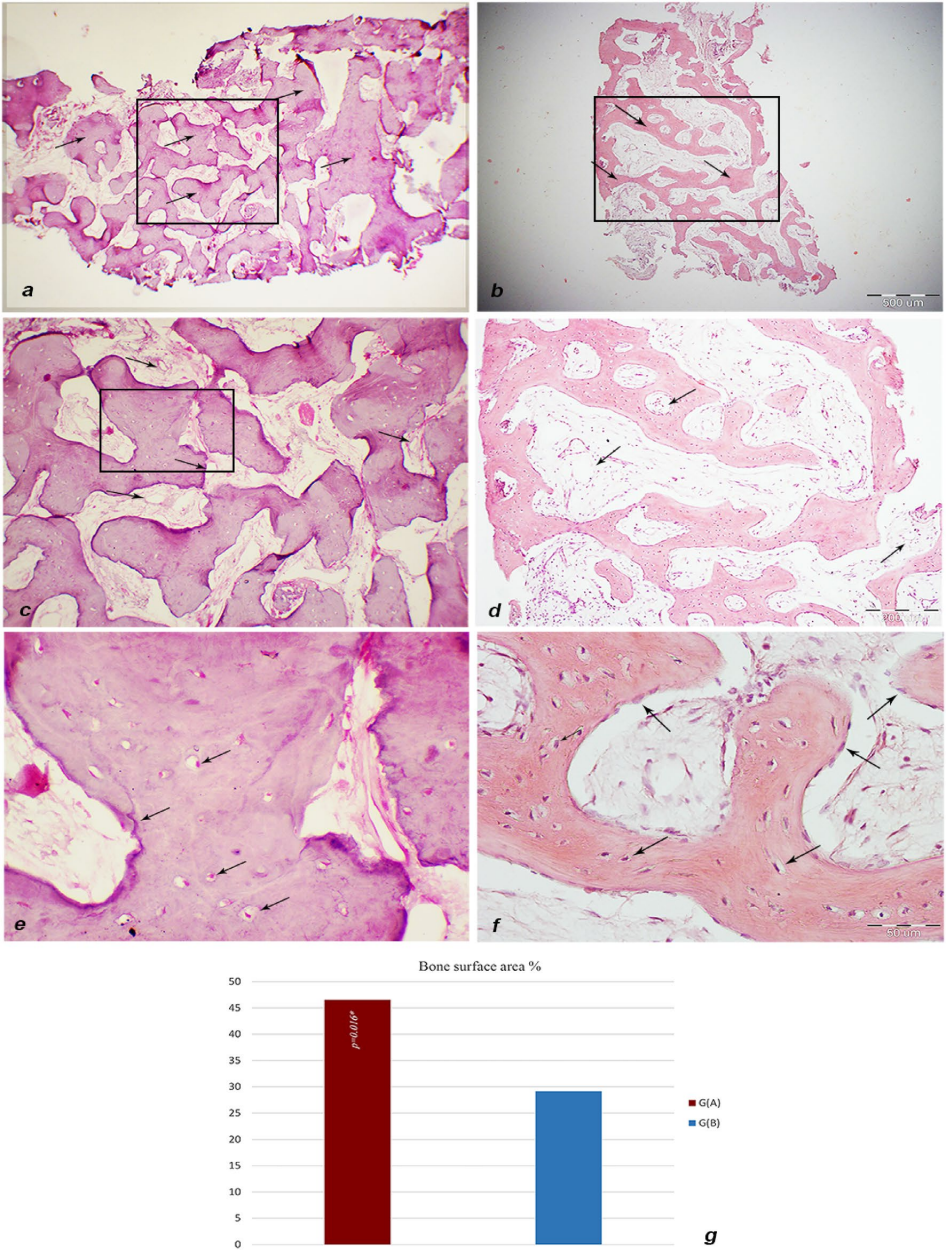
Light micrograph (LM) of H&E stained bone samples of both groups: (**a**) Group A sample showing numerous cancellous bone trabeculae. (**b**) Group B sample showing thinner cancellous bone trabeculae. (**c**) Higher magnification of group A sample micrograph inset showing the thickness of bone trabeculae surrounding bone marrow spaces containing blood vessels (arrows). (**d**) Higher magnification of group B sample micrograph inset showing bone trabeculae surrounding bone marrow spaces containing blood vessels (arrows). (**e**) Higher magnification of group A sample micrograph inset showing bone trabeculae surrounding bone marrow spaces containing blood vessels (arrows). (**f**) Higher magnification of group B sample showing osteocytes (short arrows), and osteoblasts (long arrows). (**a** & **b**) ×40, (**c** & **d**) ×100, (**e** & **f**) ×400. (**g**) Histomorphometric analysis result graph showing significantly higher bone surface area percentage in group (A) (Red 650 nm Diode).

Light microscope examination of samples of Group (A) revealed the formation of mature cancellous bone trabeculae containing regularly distributed osteocyte lacunae (Fig. 9a–c). On the other hand, thinner cancellous trabeculae were observed in group (B) (Fig. 9d–f).

#### Masson–Goldner Trichrome stained sections

**Figure 10:**
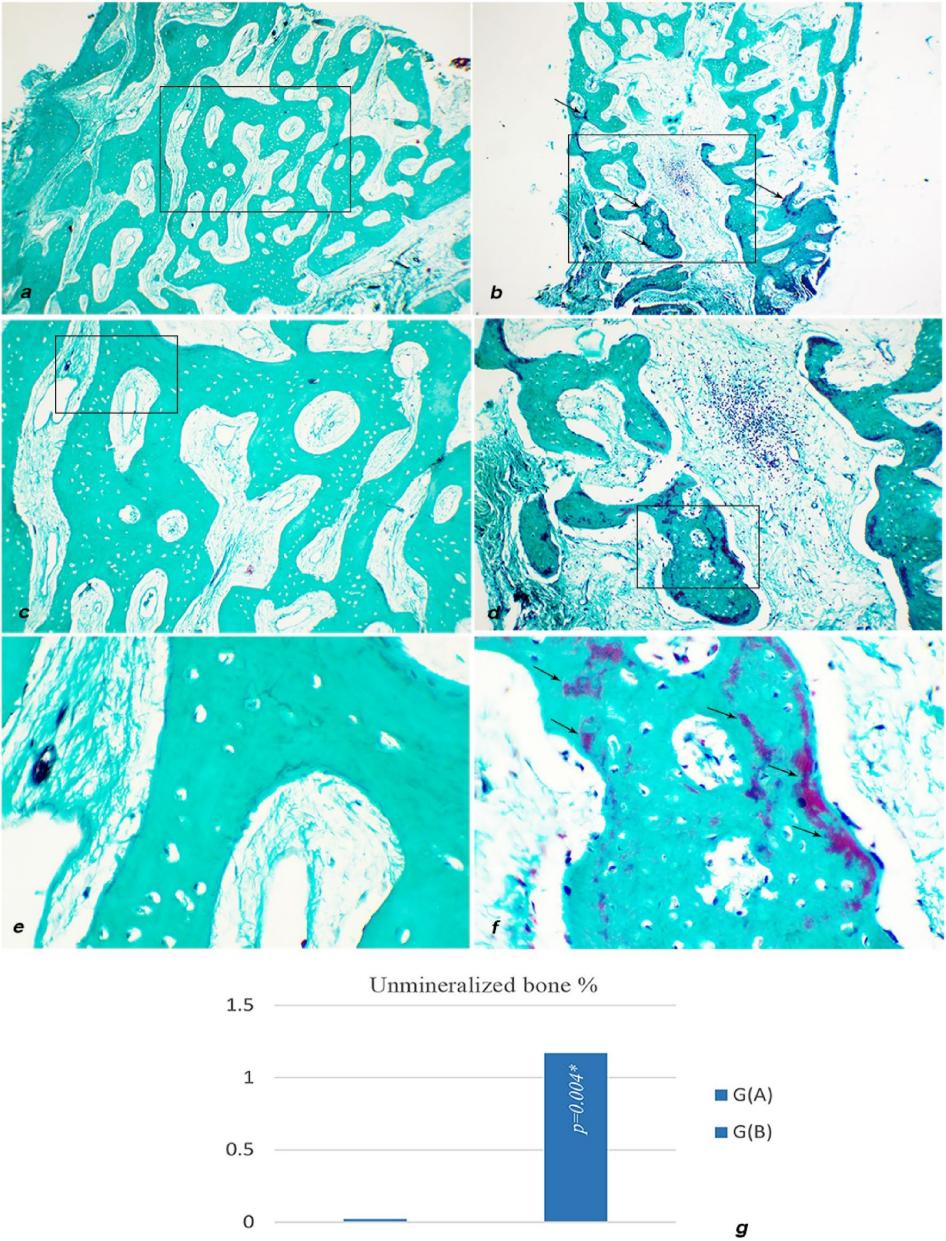
Light micrograph (LM) of Masson–Goldner Trichrome stained bone samples of both groups: (**a**) Group A sample showing mineralized cancellous bone trabeculae. (**b**) Group B sample showing few scattered areas of unmineralized bone (stained red) (arrows). (**c**) Higher magnification of group A sample micrograph inset. (**d**) Higher magnification of group B sample micrograph inset. (**e**) Higher magnification of group A sample micrograph inset showing the homogeneity of bone mineralization (stained green). (**f**) Higher magnification of group B sample micrograph inset showing unmineralized areas of bone. (**a** & **b**) ×40, (**c** & **d**) ×100, (**e** & **f**) × 400. (**g**) Histomorphometric analysis result graph showing significantly higher areas of unmineralized bone percentage in group B (Infrared 810 nm Diode).

Both groups showed homogenously mineralized bone. However, minor areas of unmineralized bone (stained red) were more observed in Group B (Fig. 10).

### Histomorphometric analysis (Table 3)

**Table 3:** Comparison between the two studied groups according to histomorphometric analysis of percentage of both newly formed bone surface area and unmineralized bone.

Histomorphometric analysis	Group A (Diode 650 nm)	Group B (Diode 810 nm)	Test of significance *p value*
Newly formed bone surface area (%)			
Min.–Max	21.417–66.733	12.760–42.598	Z_(MW)_ = 2.041
Median	47.908	29.984	*p* = 0.041*
95% CI of the median	28.368–64.510	23.155–36.246	
Unmineralized bone surface area (%)			
Min.–Max	0.003–0.081	0.161–2.798	Z_(MW)_ = 3.780
Median	0.018	1.010	*p* < 0.001*
95% CI of the median	0.008–0.028	0.301–1.872	

Min–Max: Minimum–Maximum, CI: Confidence interval, MW: Mann–Whitney U test, *: Statistically significant (*p* < 0.05).

- Comparison between both study groups has shown that newly formed bone surface area percentage in group (A) specimens was significantly higher than group (B) *p* = 0.041. (Fig. 9g)
- Upon evaluation of unmineralized bone surface area, Group (B) has demonstrated a statistically significant elevation in percentage of unmineralized bone areas compared to Group (A) *p* < 0.001 (Fig. 10g)

### Immunohistochemical investigations: (Table 4)

**Table 4:** Comparison between the two studied groups according to histomorphometric analysis of OCN reaction area % & optical density.

Immunohistochemistry	Group A (Diode 650 nm)	Group B (Diode 810 nm)	Test of significance *p value*
OCN reaction surface area (%)			
Min.–Max	5.821–17.688	2.162–6.517	Z_(MW)_ = 3.402
Median	8.738	4.27	*p* = 0.001*
95% CI of the Median	6.200–10.758	4.270–3.205	
OCN optical density			
Min.–Max	1.377–1.503	1.267–1.339	Z_(MW)_ = 3.780
Median	1.430	1.306	*p* < 0.001*
95% CI of the Median	1.402–1.479	1.274–1.325	

Min–Max: Minimum–Maximum, CI: Confidence interval, MW: Mann–Whitney U test, *: Statistically significant (*p* < 0.05).

**Figure 11:**
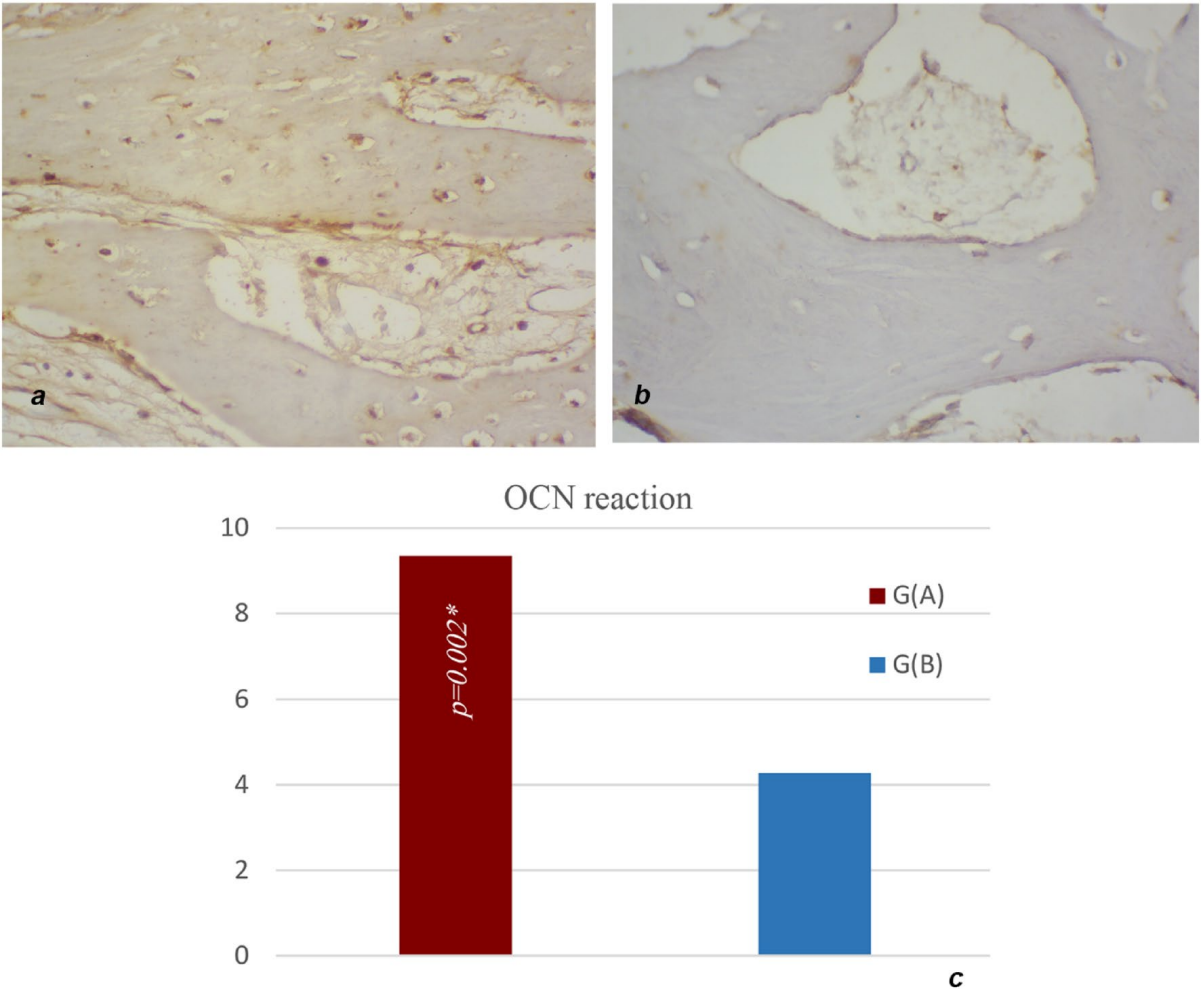
Immunohistochemical sections representing Osteocalcin reaction: (**a**) Bone sample group A vs. (**b**) Bone sample group B. (**a** & **b**) ×400. (**c**) Graph showing significantly higher OCN reaction area percentage in group A (650 nm Diode).

Both immunohistochemical parameters; Osteocalcin reaction area percentage & optical density, have significantly leaned towards the red laser (650 nm) or Group A where p < 0.001. (Fig. 11).

## Discussion

Ideal post extraction alveolar socket healing is a crucial goal of dental implant practitioners. From a surgical standpoint, better healing equals less post-operative complications. Whereas, prosthodontists aim for optimal healing in order to continue with the restorative phase of the rehabilitation procedure^10,58^.

Alveolar socket preservation with laser Photobiomodulation (PBM) assistance is currently a proven technology that has gained popularity over the typical uninterrupted healing procedure^59^. What attracts our attention, however, is the general lack of consensus among earlier investigations about the appropriate laser settings^60–63^.

To get a clear conclusion in this regard, this clinical experiment was created. The best wavelength for alveolar socket PBM was investigated by comparing two distinct lasers in the same study; a visible (red) 650 nm Diode laser and an invisible (near infrared) 810 nm Diode laser.

An inexpensive and convenient biomaterial for socket preservation is collagen-based plugs. As it has been proven to help to maintain the ridge dimensions after tooth extractions. Advantages of collagen sponge could be summarized by its high biocompatibility and full, but slow, resorption, thanks to its cross-linked structure^18^.

Regarding the laser wavelengths chosen, our intention was to use different laser families, i.e. visible vs. invisible lasers. As a consequence, each laser would offer a plausible depiction regarding the expected outcome of using its group. Surprisingly, the 650 nm Diode isn't all that common in the area of PBMT in humans, despite its demonstrated efficacy in animal studies^30–32^. Since the 810 nm Diode has had extensive testing^36,37,40^ as a reliable PBMT source, we decided that it should serve as the opposite arm in our clinical study.

Smaller tips previously used required multiple point applications for buccal and lingual surfaces, which complicates tip placement. In the current work, the 6 mm-diameter fiber optic was already meant to flawlessly adjust to the molar extraction sockets from either the occlusal, buccal, or lingual surfaces. However, because to the impingement of nearby teeth, greater diameters may prevent occlusal surface irradiation^36,37^.

Larger optical spots have shown superior clinical results for shallow and sub-surface therapy as compared to smaller spot sizes. This is due to the way they account for the laser beam's attenuation in deep tissue after energy scatter^63^.

Energy density is a key radiation parameter for PBMT; in our investigation, the length of irradiation was changed in proportion to power and spot area (63 J/cm^2^) to imitate the least effective value found in the literature, which was 50 J/cm^2^^40^. Also, in order to achieve higher energy density, longer irradiation times would be necessary, which can be uncomfortable for the patient because it requires a wider mouth opening to operate on the posterior jaw.

We chose to start PBM courses immediately as the dental extraction was finished since PBM treatment, either alone or in conjunction with other therapies, has considerable impact on the early phases of tissue recovery. The fact that PBM treatment is more successful than any particular time period provided support for this approach. In the same perspective, since it has been widely established that numerous exposures spaced regularly outperform single doses^64–67^. The sessions we planned were intended to suit most patients. The patients' presentation the day after the extraction, followed by appointments every 48 h for a week, and a final session 1 week later, for a total of six visits in the two weeks after the extraction, was not too difficult.

Recalling patients for assessment after three months seemed reasonable for two reasons: first, to provide a detailed picture of the socket healing process; and second, to enable optimum implant design selection and placement^68^.

Cone beam computed tomography (CBCT) is a cost-effective and practical pre-implant diagnostic technique. Such radiographic method has been proved to be accurate and reliable in measuring alveolar bone dimensions^69^.

In order to locate specific antigens in cells and tissue, immunohistochemistry (IHC) uses the specific binding between an antibody and antigen. This method is most frequently used to identify and study specific antigens under a light microscope^70^.

Bone histomorphometry is a unique tool that is generally applied to images of histological sections to provide a quantitative assessment of bone remodeling, modeling, and structure^71^. In this work, it was employed to measure the percentage of new bone surface area, unmineralized bone, immunonistochemical marker reaction area percentage, as well as its optical density.

In this study, CBCT scans analysis showed that both lasers managed to positively affect alveolar ridge surface area, which in turn refers to the fact that applied technique has exceeded expectations, which was to save the bony architecture from collapsing. Achieving larger bone volume without using bone substrate grafting material may point to the definite impressive role of PBMT in the process of bone repair.

On top of that, the infrared Diode (810 nm) appeared to surpass its visible counterpart in this regard as the dimensional change between immediately post extraction and 3 months post operatively was found to be statistically significant.

In the same context, Rosero^36^ and Romao^37^ have used the 808 nm Diode as a PBM source for alveolar socket preservation and proved higher bone volume compared to control.

According to our search, no human study has evaluated alveolar bone dimensions after laser PBM using 650 nm Diode.

Regarding histomorphometric analysis of bone samples, the red laser (650 nm) showed a significantly higher bone trabecular surface area percentage than the 810 nm infrared laser. On top of that, statistically significant lesser areas of unmineralized bone were observed with this group; the 650 nm laser, indicating, faster regenerative process than the infrared laser.

Monea et al.^39^ have tested the effect of red visible LED light PBM and noticed formation of abundant bone trabeculae compared to a control group. In addition, they proposed that tested PBMT technique has demonstrated higher rate of bone regeneration than the other group.

On the other hand, the near infrared laser (810 nm) PBM has managed to demonstrate similar results according to Rosero^36^ and Romao^37^, one more time, when tested against natural uninterrupted extraction socket healing (control).

Furthermore, animal studies as well document the encouraging effect of PBMT on bone repair, with elevated trabeculae and bone mineral density, plus the mechanical properties of the regenerated bone^72,73^.

Mature osteoblasts produce the small protein osteocalcin (OCN), which is mostly deposited in the extracellular matrix of bone. According to reports, OCN may have several roles in bone metabolism. It is a key player in bone remodeling, having an impact on both osteoblast and osteoclast activity, and it also regulates bone mineralization^74^.

Regarding immunohistochemical examination in this study, once again, the red laser got an edge over the infrared one as the area percentage of expression of bone marker Osteocalcin, plus, its optical density in bone samples has significantly leaned towards Group A.

Our results come to an agreement with multiple in vitro^75–77^ and in vivo^78–81^ animal studies that validated the effect of low-level laser on the expression of Osteocalcin.

### Limitations of the study

Patients of same gender who fall under the same age group like the ones recruited in this study, still differs in healing capacity, therefore, we think that a split mouth study design may have been more reliable when it comes to assessing healing parameters, this way, fixed conditions are assured, and consequently, more accurate outcome.

Mandibular molars represent the most vulnerable group of teeth in the oral cavity, making them in more need of research. However, the natural anatomy of such multirooted teeth with the presence of inter radicular septum, may be considered a source of error in histological findings, because when the septum is high enough and somehow survived during extraction, the sample collected with the trephine bur could quite represent the septum, not the intended healing socket. According to our perspective, single rooted teeth may serve better when histological examination of extraction socket healing is considered.

## Conclusion

The fact that the infrared laser (810 nm) had the edge over the red laser (650 nm) concerning alveolar bone surface area traced on CBCT, coupled with the detected superiority of the red Diode across the histological parameters, may hint to the higher quality of newly formed bone with the (650 nm) red laser PBMT while the greater alveolar ridge dimensions may be achieved using the Infrared Diode.

Generally, we would rather conclude from this work that the red 650 nm Diode needs to attract more attention of future extraction socket photobiomodulation trials as, like it was mentioned earlier, this wavelength is poorly presented in the literature concerned with alveolar socket PBM in humans.

### Clinical relevance

This study belongs to the next wave of studies that tackle the subject of photobiomodulation assisted alveolar bone regeneration. Earlier batch of trials were meant to prove the superiority of photobiomodulation over the regular uninterrupted healing. We thought that it's time to dig a little deep and decide best PBMT parameters to do so.

First phase in this regard is deciding best source for photobiomodulation therapy, or in other words, the best laser wavelength. Since we are concerned with socket preservation, so our main clinical relevance would be socket dimensions or surface area of the extraction socket intended to receive implant. Referring to the outcome of this work, we could claim that we managed to draw some interest to the red (650 nm) laser as a target for future trials.

From the authors point of view, future progress would be to cement a certain wavelength as a preferred source for PBMT socket preservation, and then proceed to determine the optimum irradiation parameters (mainly energy density) and finally, number and distribution of required sessions that would benefit the healing process and, in the same time, suit regular patients' schedule. This way, clinicians would have an applicable evidence based protocol for socket preservation PBMT.

These results may point to the value of red (650 nm) Diode which is almost neglected in regard to extraction socket regeneration, and hopefully, attract future projects to extensively investigate this laser’s role in regenerative dentistry.

## Data Availability

The datasets used and analyzed during the current study are available from the corresponding author on reasonable request.
